# *Redondoviridae*: High Prevalence and Possibly Chronic Shedding in Human Respiratory Tract, But No Zoonotic Transmission

**DOI:** 10.3390/v13040533

**Published:** 2021-03-24

**Authors:** Nguyen Thi Kha Tu, Xutao Deng, Nguyen Thi Thu Hong, Nguyen Thi Han Ny, Tran My Phuc, Pham Thi Thanh Tam, Duong An Han, Luu Thi Thu Ha, Guy Thwaites, H. Rogier van Doorn, Anna-Maija K. Virtala, Eric Delwart, Stephen Baker, Olli Vapalahti, Le Van Tan, on behalf of the VIZIONS Consortium

**Affiliations:** 1 Doctoral School in Health Sciences, Faculty of Medicine, University of Helsinki, 00014 Helsinki, Finland; olli.vapalahti@helsinki.fi; 2 Emerging Infection Group, Oxford University Clinical Research Unit, Ho Chi Minh City 7000, Vietnam; hongntt@oucru.org (N.T.T.H.); nynth@oucru.org (N.T.H.N.); phuctm@oucru.org (T.M.P.); tamptt@oucru.org (P.T.T.T.); gthwaites@oucru.org (G.T.); 3 Dong Thap Provincial Center for Disease Control, Cao Lanh City 660273, Dong Thap Province, Vietnam; anhanduong@gmail.com (D.A.H.); luuthithuha2018@gmail.com (L.T.T.H.); 4 Department of Laboratory Medicine, University of California, San Francisco, CA 94143, USA; XDeng@bloodsystems.org (X.D.); Eric.Delwart@ucsf.edu (E.D.); 5 Vitalant Research Institute, San Francisco, CA 94118, USA; 6 Centre for Tropical Medicine and Global Health, Nuffield Department of Medicine, University of Oxford, Oxford OX3 7LG, UK; rvandoorn@oucru.org; 7 Oxford University Clinical Research Unit, Ha Noi 8000, Vietnam; 8 Department of Veterinary Biosciences, Faculty of Veterinary Medicine, University of Helsinki, 00014 Helsinki, Finland; anna-maija.virtala@helsinki.fi; 9 Cambridge Institute of Therapeutic Immunology & Infectious Disease (CITIID), Department of Medicine, University of Cambridge, Cambridge CB2 0QQ, UK; sgb47@medschl.cam.ac.uk; 10 Virology and Immunology, HUSLAB, Helsinki University Hospital, 00029 Helsinki, Finland

**Keywords:** redondoviruses, vientovirus, brisavirus, persistence, respiratory, animals, zoonosis

## Abstract

*Redondoviridae* is a recently discovered DNA virus family consisting of two species, *vientovirus* and *brisavirus*. Here we used PCR amplification and sequencing to characterize redondoviruses in nasal/throat swabs collected longitudinally from a cohort of 58 individuals working with animals in Vietnam. We additionally analyzed samples from animals to which redondovirus DNA-positive participants were exposed. Redondoviruses were detected in approximately 60% of study participants, including 33% (30/91) of samples collected during episodes of acute respiratory disease and in 50% (29/58) of baseline samples (with no respiratory symptoms). Vientovirus (73%; 24/33) was detected more frequently in samples than brisaviruses (27%; 9/33). In the 23 participants with at least 2 redondovirus-positive samples among their longitudinal samples, 10 (43.5%) had identical redondovirus replication-gene sequences detected (sampling duration: 35–132 days). We found no identical redondovirus replication genes in samples from different participants, and no redondoviruses were detected in 53 pooled nasal/throat swabs collected from domestic animals. Phylogenetic analysis described no large-scale geographical clustering between viruses from Vietnam, the US, Spain, and China, indicating that redondoviruses are highly genetically diverse and have a wide geographical distribution. Collectively, our study provides novel insights into the *Redondoviridae* family in humans, describing a high prevalence, potentially associated with chronic shedding in the respiratory tract with lack of evidence of zoonotic transmission from close animal contacts. The tropism and potential pathogenicity of this viral family remain to be determined.

## 1. Introduction

Acute viral respiratory infections are associated with a significant global disease burden and are associated with the majority of epidemics and pandemics [1,2], including the ongoing SARS-CoV-2 pandemic [3]. Often, the etiological agent in the majority of the patients presenting with acute respiratory infections remains undetermined [4,5,6,7]. Therefore, it is critical to assess the potential clinical significance of newly discovered viruses, particularly to inform clinical management and health policymakers.

*Redondoviridae* is a novel virus family within the circular Rep-encoding single-stranded (CRESS) group of DNA viruses [8]. This family consists of only one genus, *Torbevirus*, which is divided into two species, vientovirus and brisavirus. The accepted species demarcation is ≤50% sequence similarity of the replication protein [8,9].

Redondoviruses have been exclusively detected in samples from humans, especially those collected from the respiratory tract [8,10,11,12]. Redondovirus DNA was detected in 15% (9/60), 11% (22/209), and 2% (2/100) of oropharyngeal samples taken from healthy adults in the US [8], Italy [10], and Spain [11]. Higher loads of redondovirus DNA were detected in respiratory samples from critically ill patients than in those from healthy individuals [8]. Redondoviruses may also be associated with periodontal disease because their abundance was noted to decrease with standard periodontal treatment [8]. Moreover, persistent detection of redondoviruses in serial endotracheal aspirates from critically ill subjects over 2–3 weeks has been documented [8].

Existing data suggest that redondoviruses are unlikely to be bacteriophage because they carry no prokaryotic ribosome binding site [8]. There is currently no evidence regarding the targeted detection of redondoviruses in animals, fresh water, marine, air, or soil samples [8]. Screening is generally performed via metagenomic sequence analysis, but PCR amplification remains the gold standard for the targeted detection of microbes. Additionally, data regarding the host range, prevalence, and key characteristics of this recently discovered virus family remain scarce.

Collectively, given the pathogenic potential of redondoviruses, as well as existing knowledge gaps regarding their epidemiology and evolution, we aimed to investigate their genetic diversity, epidemiological features, and potential for zoonotic transfer. These data might aid the prioritization of appropriate intervention strategies in the future.

## 2. Materials and Methods

### 2.1. The High-Risk Sentinel Cohort Study

Samples from this investigation were derived from a previously described cohort study conducted in Vietnam [6,13]. In brief, the cohort comprised healthy individuals working with animals in Dong Thap Province (*n* = 282) and Dak Lak Province (*n* = 299) in Mekong Delta and central highlands of Vietnam, respectively. Recruitment was initiated in March 2013 in Dong Thap Province and from February 2014 in Dak Lak Province. The study participants were followed for 3 years (4/2013–4/2016 for the Dong Thap site and 2/2014–2/2017 for the Dak Lak site).

We collected respiratory samples (nasal and throat swabs) from the participants and their animals at the beginning of each year when no respiratory symptoms were present. These samples were defined as baseline samples. Over the 3-year follow-up period, we collected disease-episode samples from the diseased participants and their animals whenever the participants reported they had an acute respiratory infection. Acute respiratory infection was defined as any signs/symptoms of respiratory tract infections with fever (≥38 °C).

Here we focused on nasal/throat swabs collected during all respiratory disease episodes reported in 2013 (*n* = 91). These samples were collected from 58 study participants residing in Dong Thap Province. Additionally, all baseline samples (*n* = 58) of these participants were analyzed. To assess the zoonotic potential of detected redondovirus, we tested nasal/throat swabs collected from animals to which the redondovirus-positive participants (farmers) were exposed during each specific disease episode.

### 2.2. Whole-Genome Amplification by Inverse PCR

The complete viral genome was amplified by inverse PCR using specific primers (Table 1) designed from metagenomic contigs. The PCR was conducted in a final 25 μL volume reaction mixture, containing 18 μL of Platinum™ PCR SuperMix High Fidelity (Invitrogen, Carlsbad, CA, USA), 1 μL of each reverse and forward primer at a concentration of 10 μM each, and 5 μL of extracted nucleic acid. PCR reactions were performed using a Mastercycler (Eppendorf, Hamburg, Germany) (Table 1).

Additionally, we employed a primer-walking strategy to close gaps within the genomes (Table 1). PCR amplicons were detected using 1% agarose gels and sequenced using a BigDye Terminator v1.1 cycle sequencing kit (Applied Biosystems, Carlsbad, CA, USA) on an ABI377 automatic sequencer (Applied Biosystems), following the manufacturer’s instructions.

To minimize the likelihood that vientovirus sequences were derived from nucleic acid extraction kits, which has been previously reported [14,15], we used 2 nucleic acid extractions. One source was newly extracted from the original sample using a MagNApure 96 platform (Roche Diagnostics, Mannheim, Germany) [13]. The other comprised residual nucleic acid materials after mNGS sequencing extracted by the QIAamp 96 Virus QIAcube HT Kit (QIAGEN GmbH, Hilden, Germany) [16].

### 2.3. PCR Screening and Genetic Characterization of Redondoviruses in Respiratory Samples and Animal Contacts

We used residual nucleic acid extractions from human disease-episode samples [13] for the PCR screening of redondoviruses. We extracted nucleic acid using the MagNApure 96 platform. For samples collected at baseline or from animals, nucleic acid was freshly isolated from the original materials using the QIAamp viral RNA kit (QIAgen GmbH, Hilden, Germany), following the manufacturer’s instructions.

To investigate the prevalence of redondoviruses in human and animal samples, we employed a generic single-round PCR assay targeting a conserved region of the capsid protein-coding gene. The primer sequences are described in Table 1.

To genetically characterize the amplified redondovirus nucleic acid, we applied a generic PCR to amplify the entire replication protein-coding gene in samples positive by the capsid-gene PCR (Table 1). The PCR primers were newly designed from the complete genome generated as part of the initial experiment described above and available redondovirus sequences deposited in the GenBank [8].

We used Sanger sequencing to sequence the generated PCR amplicons. The PCR and sequencing procedures used were comparable to those used for confirmatory PCR and sequencing above, with some modifications to the thermal cycling conditions (Table 1). Negative controls were included in each PCR detection experiment. The PCR-associated experiments were conducted in unidirectional molecular diagnostic facilities consisting of three physically separated laboratories for reagent preparation, nucleic acid extraction, and amplification to minimize the risk of contamination.

### 2.4. Phylogenetic Analysis

Sequence alignments were conducted in MUSCLE available in MEGA version X. Phylogenetic trees were constructed using the generated nucleotide for genetic characterization using the Maximum Likelihood method available in the MEGA software with a bootstrap value of 1000 replicates.

### 2.5. Nucleotide Sequence Accession Numbers

The redondovirus genomes and replication coding sequences described here were submitted to GenBank under the Accession Numbers MT759843, MT823476–MT823478, and MW216334–MW216337.

### 2.6. Statistics

Statistical associations and differences between variables were calculated using Pearson’s Chi-squared test or Fisher’s exact test for categorical data and t-test for continuous data, respectively, by pairwise comparisons in STATA software (version 12.0). *p*-values were adjusted for multiple comparisons by the Benjamini and Hochberg method [17] with a false discovery rate (FDR) calculator [18]. A value of *p* ≤ 0.05 was considered significant.

### 2.7. Ethics

The high-risk sentinel cohort study received approvals from the Ethics Committees at the University of Oxford, United Kingdom, and at the sub-Departments of Animal Health and General Hospital in Dong Thap Province and Dak Lak Province and in the Hospital of Tropical Diseases in Ho Chi Minh City in Vietnam, as reported previously [16,19]. Written consent was obtained from each study participant.

## 3. Results

### 3.1. Detection and Genetic Characterization of a Vientovirus

**Figure 1:**
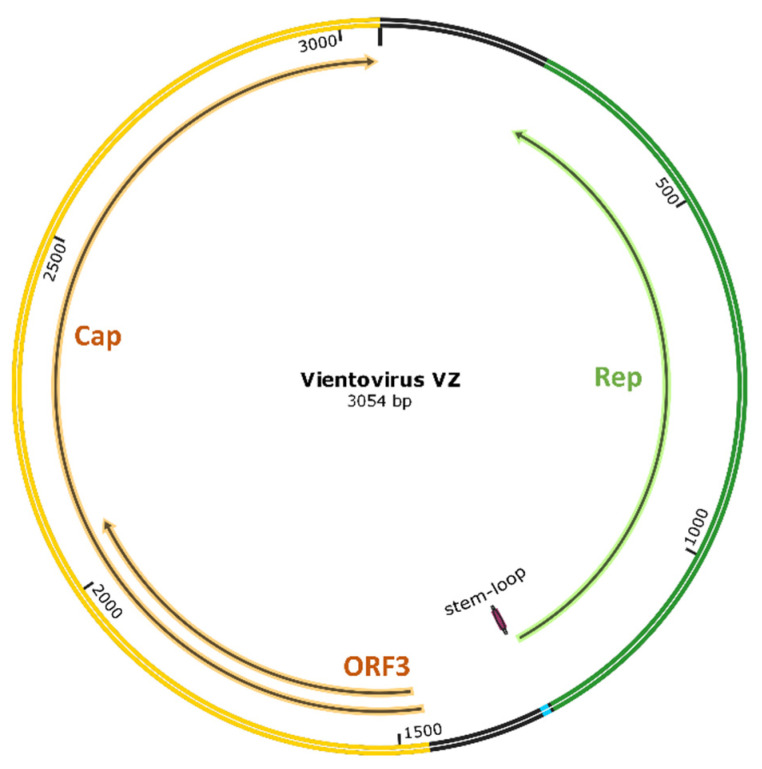
Putative genome organization of vientovirus VZ. Vientovirus VZ has typical genome features of a virus of the *Redondoviridae* family. Cap: capsid protein; Rep: replication protein; ORF3: open reading frame 3 encoding an unknown protein.

We previously detected a contig derived from two reads related to the human lung-associated vientovirus AL strain (Accession Number: MK059760.1) in one sample using metagenomic sequencing [16]. Using inverse PCR, we recovered a full circular genome of this virus, which was 3054-bp. A sequence comparison found that the generated sequence was closely related to the reported genomes of vientovirus of the family *Redondoviridae* (sharing a 79% sequence identity (2404/3054 bp)). The obtained sequence possessed a typical genomic structure of this viral family, containing three open reading frames (ORF1-3) encoding for capsid, replication, and a protein of unknown function (530, 350, and 200 AA, respectively). The coding region of the capsid protein and the protein of unknown function was arranged in an opposite orientation to the replication protein (Figure 1). Additionally, a typical stem-loop structure (“TATTATTTAT”) was identified upstream of the 5′ end of the replication protein-coding region (Figure 1).

**Figure 2:**
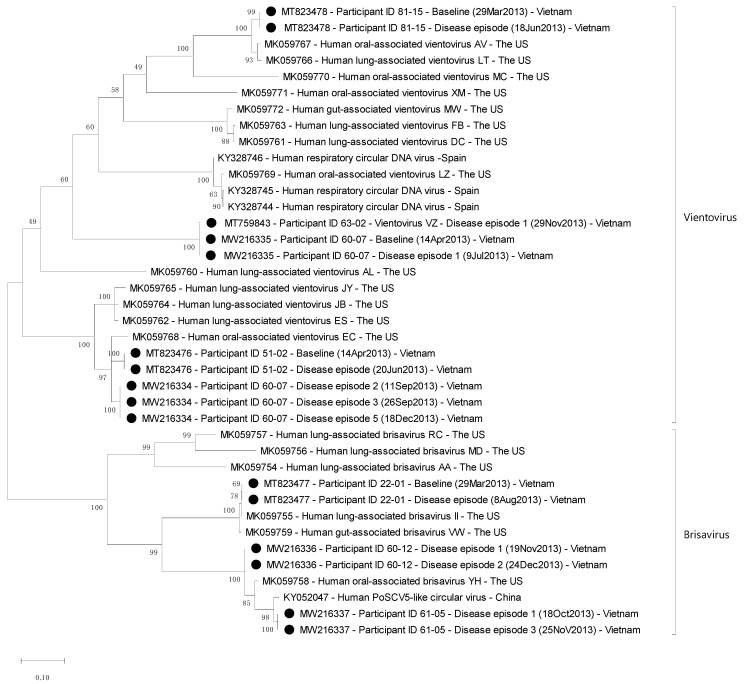
Phylogenetic tree of complete nucleic acid sequences of the replication protein-coding gene of redondoviruses. The sequence Accession Numbers are included on the tips of the tree. Black circles denote redondovirus strains detected in the present study.

A pairwise comparison demonstrated that the capsid and replication protein sequences share the highest similarity (97.7% and 59.1%) with respective protein sequences (Accession Numbers: QCD25327.1 and QCD25302.1, respectively), corresponding with 98.1% and 66.6% of similarities at the nucleotide level of the vientovirus (Accession Numbers: MK059768 and MK059760). Phylogenetic analysis of replication-gene nucleic acid showed a close relatedness with previously reported vientovirus sequences (Figure 2). The detected virus was confirmed as vientovirus, which we named vientovirus VZ (Accession Number: MT759843).

### 3.2. Detection of Redondoviruses in Respiratory Samples

We performed subsequent PCR screening and detected redondovirus DNA in 29 of 58 (50%) baseline samples from 58 participants (Table 2). We additionally detected redondovirus DNA in 30/91 (32.7%) disease-episode samples from the same participants (Table 2).

Overall, after combining the data from the baseline and disease-episode samples, we detected redondoviruses in at least one longitudinal sample collected at baseline and disease episodes in over half of the participants (33/58; 56.9%) (Table 2).

Sequencing of the PCR amplicons was successful in 26/29 and 27/30 positive samples at baseline and during disease episodes, respectively. Of the 26 sequences obtained from the baseline samples, 6 (23.1%) belonged to brisavirus, and 20 (76.9%) belonged to vientovirus (Table 2). Of the 27 sequences obtained from the disease-episode samples, 9 sequences (33.3%) belonged to brisavirus, and 18 (66.7%) belonged to vientovirus (Table 2).

### 3.3. The Genetic Diversity of Redondoviruses

We next compared 16 complete replication protein-coding sequences of redondoviruses that we obtained in the present study with those isolated from the US, Spain, and China available in GenBank. A pairwise comparison and phylogenetic analysis revealed that there was no extensive geographical clustering among viruses detected in Vietnam, the US, Spain, and China (Figure 2).

### 3.4. Evidence of Possible Persistence of Redondoviruses in Nasopharynx

Of the 23 participants with at least two longitudinal samples that were positive for redondoviruses, 10 (43.5%) provided evidence of having an identical replication gene of redondovirus (610–1306 bp, equivalent to 58–100% of complete nucleic acid sequence coding replication protein) detected in their longitudinal samples within a window of 35–132 days (Table 3). In one patient (ID 60-07), we detected vientovirus VZ with the same replication protein-coding gene in nasal/throat swabs collected at baseline and disease episode No. 1. However, in subsequent disease episodes, a genetically related but nonidentical vientovirus was detected (Table 3).

### 3.5. The Demographics of Participants with and without Redondoviruses Detected in at Least One of Their Longitudinal Samples Taken at Baseline and Disease Episodes

The demographics of the 58 study participants with redondoviruses detected in at least one of their serial samples at both baseline and disease episodes are presented in Table 4. Notably, the redondovirus-positive participants were significantly older than those negative for redondoviruses (43.8 vs. 33.8, *p* = 0.02) (Table 4). The participants were more likely to test positive for redondoviruses if their occupation was a slaughterer (45.5% vs. 15%, *p* = 0.02) (Table 4).

### 3.6. Clinical Symptoms of Redondovirus-Infected Patients during Disease Episodes

Coughing was the most common clinical symptom recorded in the redondovirus-infected patients, followed by sneezing and a sore throat. Dyspnea and watery diarrhea were recorded in 10% (3/30)) and 13% (4/30) of the participants, respectively. There was no significant difference in respiratory symptoms between individuals with and without a redondovirus detected in respiratory samples (*p* = 0.24; (Table 5)). Likewise, there was no significant difference in clinical symptoms between the brisavirus- and vientovirus-positive participants (Table 5).

### 3.7. Coinfection in Samples Having Redondoviruses Detected with Other Respiratory Viruses

Taking into account the results of our previous PCR screening [13] and mNGS analysis [16], we identified a mixed infection of redondoviruses and other viruses in 28 samples. The codetected viruses included gemycircularvirus VIZIONS-2013, cyclovirus VIZIONS-2013, human rhinovirus, statovirus VIZIONS-2013, RSV A, gemycircularvirus, enterovirus, statovirus, and influenza A virus (Table 6).

### 3.8. Detection of Redondoviruses in Respiratory Samples of Animals

We screened 27 samples from 27 pigs from 5 households, 13 pooled samples from 27 chickens from 5 households, 8 pooled samples from 17 Muscovy ducks from 2 households, 1 sample from a duck, and 4 pooled samples from 6 dogs from 4 households for redondovirus by generic PCR. None tested positive.

## 4. Discussion

Here we report the detection and genetic characterization of several redondovirus species of the recently discovered *Redondoviridae* family [8,12] in longitudinal upper respiratory tract samples of individuals at potential risk of zoonotic disease exposure and their animal contacts [6]. We found that nearly 60% of tested human participants were positive for either brisavirus or vientovirus of the family *Redondoviridae*, while none of the animals tested were positive for these viruses; these data are largely in agreement with a previous report [8]. Notably, we identified the same redondovirus replication protein-coding gene in longitudinal samples of 10 participants for up to 5 months. In a previous study, redondovirus DNA was detectable in serial samples collected from several patients over 2–3 weeks [8]. Collectively, these data suggest the persistence of the redondoviruses in the human respiratory tract, although sequence comparison at the whole-genome level is needed to confirm the relatedness between these redondovirus strains. Collectively, this study provides additional evidence supporting the possibility that redondoviruses, or their host(s) if not human cells, can colonize the human respiratory tract. Therefore, their pathogenic potential for humans warrants further research.

The prevalence (56.9%) of redondoviruses detected in our study participants was higher than the reported prevalence of 15% in the oropharynx of healthy Americans [8], 11% among Italians [10], and 2% among Spanish subjects [11]. However, phylogenetic analysis found no large-scale geographical clustering between viruses detected in Vietnam, the US, Spain, and China, indicating the wide geographic distribution and genetic diversity of redondoviruses.

Additionally, we observed a higher proportion of redondoviruses detected in samples at baseline than during disease episodes of the study participants. However, higher copy numbers of redondovirus DNA were previously reported in oropharyngeal samples of critically ill patients versus those of healthy individuals [8]. Thus, future studies should assess the kinetics of redondoviral loads over the course of the illness as well as between disease episodes and at baseline.

This work represents the first PCR screening study for redondoviruses in domestic animals from one of the recognized global hotspots of emerging infections. The sampled domestic animals were from households of study participants who tested positive for redondovirus. We found no evidence for redondoviruses in the respiratory tracts of these domestic animals. The absence of redondovirus in animal samples is in line with a recent report that used metagenomics [8]. The data also suggest that cross-species transmission was unlikely to occur among our study subjects. However, sequences of CRESS-DNA viruses have been widely found in animals [20,21]. More recently, deltaviruses that were theoretically confined to humans were detected in birds, snakes, fish, amphibians, and invertebrates [22,23]. Notably, we found that redondovirus-positive individuals were more likely to be animal slaughters. Therefore, whether similar or more divergent redondoviruses can be detected in animals merits further research.

Whether redondoviruses replicate in humans, other eukaryotic cellular residents of the respiratory tract, or are passively inhaled and deposited on respiratory surfaces remains unknown. An airborne environmental source seems unlikely given that closely associated animals tested PCR negative. Replication of redondoviruses in human cells also remains a possibility as a related family of CRESS-DNA viruses, the *Circoviridae*, includes members known to infect mammals [24,25].

There were no significant differences in clinical symptoms of acute respiratory illness in patients with and without redondoviruses detected in their samples, indicating, as is true for most respiratory pathogens, that clinical symptoms cannot be used to identify different etiologies. Additionally, we cannot exclude the possibility that the symptoms were caused by non-Redondoviridae viruses. Evidence for any association or causal relationship between this virus family and acute respiratory or other diseases, or lack of such association, still needs more studies; this is true also for some other newly found viruses, such as anelloviruses [8].

We found a significant difference in the detection of redondoviruses along with other respiratory viruses in this study. A previous publication demonstrated that anelloviruses were often codetected with redondoviruses [8]. Therefore, we propose the further screening of samples for redondoviruses and anelloviruses to provide a better understanding of the interaction between redondoviruses and anelloviruses.

## 5. Conclusions

Our study adds to the growing body of knowledge regarding the epidemiological features and genetic diversity of the new *Redondoviridae* family. Importantly, we found no evidence of cross-species transmission between humans and their animal contacts. Whether redondoviruses are associated with respiratory or other infections in humans requires further research.

## Figures and Tables

**Table 1:** Newly designed primer sequences for the PCRs.

Primer Name	For Purpose	Sequence	PCR Products (bp)	Target (Regions)	Thermal Cycles
Vientovirus VZ-inverse_F	Whole genome	TATTTGTGGCCTTACTCCTTGT	3000	Replication gene (2628–2649′)	95 °C for 2 m; 45 cycles of 95 °C for 15 s, 52 °C for 30 s, 72 °C for 2 m 45 s; 72 °C for 5 m
Vientovirus VZ-inverse_R	Whole genome	GGACATATAGCAGAAAAAGGTGATG		Replication gene (2577–2552′)	
Vientovirus VZ-walking_F	Whole genome	AGACTTGCTTCTATGGTTTGTAGT	1400	Capsid gene (268–291′)	95 °C for 2 m; 45 cycles of 95 °C for 15 s, 48 °C for 30 s, 72 °C for 2 m; 72 °C for 5 m
Vientovirus VZ-walking_R	Whole genome	TGATACACAATTCTTTTACCGTTGT		Capsid gene (1777–1752′)	
Vientovirus VZ-close gap_F	Whole genome	GGGGCCCTTGAACCACATTA	750	Replication gene (2352–2372′)	95 °C for 2 m; 45 cycles of 95 °C for 15 s, 52 °C for 30 s, 72 °C for 1 m 15 s; 72 °C for 5 m
Vientovirus VZ-close gap_R	Whole genome	GCAGCCCTCTTAAGCCTGTA		Replication gene (132–112′)	
Redondovirus-capsid gene_F	PCR screening	GGCTTAAGAGGGCTGCTAGG	460	Capsid gene (116–136′)	95 °C for 5 m; 45 cycles of 95 °C for 20 s, 52 °C for 30 s, 72 °C for 1 m; 72 °C for 5 m
Redondovirus-capsid gene_R		TCCTTGGATGCCATGAAACT		Capsid gene (575–555′)	
Redondovirus-replication gene_F	Genetic characterization	GTTGTCACTTGTGAAACGATGA	1400	Replication gene (1711–1733′)	95 °C for 5 m; 45 cycles of 95 °C for 20 s, 50 °C for 30 s, 72 °C for 2 m; 72 °C for 5 m
Redondovirus-replication gene_R		TCGACGATAAACTCTCTTTCTTGA		Replication gene (43–19′)	

**Table 2:** Detection of redondoviruses from the study participants and each of the baseline and clinical samples.

	Redondoviruses Negative	Redondoviruses Positive				Total
		Brisavirus	Vientovirus	Undefined *	Subtotal	
Study participants ^	25	9	23	1	**33**	58
Baseline samples	29	6	20	3	**29**	58
Disease-episode samples	61	9	18	3	**30**	91

* Redondovirus-screening PCR was positive, but no PCR sequence was obtained for species identification. ^ Number of participants who never got infected (negative) or got infected with redondoviruses at least once (positive) during the entire study are shown.

**Table 3:** Chart showing identical replication-gene sequences of brisavirus and vientovirus detected in samples at baseline and disease episodes. RedonV: redondoviruses; VienV: vientovirus; BrisaV: brisavirus. Vientovirus or brisavirus written with the same name and in the samples collected from the same participant have identical replication-gene sequences. Boxes with redondoviruses are samples positive with redondoviruses by PCRs, but no PCR-replication sequences were achieved for species identification.

	Study Year 2013						
	Baseline	Disease Episode 1	Disease Episode 2	Disease Episode 3	Disease Episode 4	Disease Episode 5	Duration of Persistence (Days)
Participant ID 60-07	VienV VZ 14-Apr	VienV VZ 09-Jul	VienV S39 11-Sep	VienV S39 26-Sep	RedonV 15-NoV	VienV S39 18-Dec	86 and 98, respectively
Participant ID 48-01	VienV S19 14-Apr	VienV S19 10-Jul					87
Participant ID 81-15	VienV S8 29-Mar	VienV S8 18-Jun					81
Participant ID 49-01	VienV S15 14-Apr	VienV S15 20-Jun					67
Participant ID 51-02	VienV S17 14-Apr	VienV S17 20-Jun					67
Participant ID 22-01	BrisaV S32 29-Mar	BrisaV S32 08-Aug					132
Participant ID 81-23	BrisaV S4 07-Apr	BrisaV S4 05-Jun	10-Jul				59
Participant ID 61-05	RedonV 14-Apr	BrisaV S56 18-Oct	RedonV 08-Nov	BrisaV S56 25-Nov			38
Participant ID 60-12	14-Apr	BrisaV S83 19-Nov	BrisaV S83 24-Dec				35

**Table 4:** The demographics of the study participants.

	Total	Redondoviruses Positive *	Redondoviruses Negative	*p*-Value
Number of participants	58	33	25	NA ^
Having chronic diseases (%)	4 (6.9)	1 (3)	3 (12)	0.3
Occupation (%)				
Animal-raising farmer	26 (44.8)	13 (39.4)	13 (52)	0.3
Animal-health worker	12 (20.7)	5 (15.2)	7 (28)	0.1
Slaughterer	18 (31)	15 (45.5)	3 (12)	0.02
Rat trader	2 (3.4)	0 (0)	2(8)	NA
Females/males (ratio)	16/42 (0.4)	11/22 (0.5)	6/19 (0.3)	1
Median age in year (range)	35.5 (7–76)	43.8 (23–76)	33.8 (7–72)	0.02#

* Number of participants who got infected with redondoviruses at least once during the entire study. ^ NA: not applicable. The value is shown in a number format (percentage). *p*-values were calculated using Pearson’s Chi-squared test or Fisher’s exact test. The *p*-values were adjusted for multiple comparisons using the Benjamini and Hochberg procedure; # by *t*-test.

**Table 5:** Clinical symptoms from 58 patients at 91 disease episodes with and without redondoviruses detected.

	No. of Disease Episodes	Redondoviruses Positive				Redondoviruses Negative	*p*-Value ^#^
		Total	Brisavirus *	Vientovirus *	*p*-Value		
	*N* = 91	*N* = 30	*N* = 9	*N* = 18	NA	*N* = 61	NA
Fever	91 (100)	30 (100)	9 (100)	18 (100)	1	61 (100)	1
Cough	75 (82.4)	24 (80)	8 (88.9)	14 (77.8)	1	51 (83.6)	1
Sneezing	69 (75.8)	22 (73.3)	5 (55.6)	15 (83.3)	0.743	47 (77.0)	1
Sore throat	49 (53.8)	19 (63.3)	5 (55.6)	13 (72.2)	1	30 (49.2)	1
Dyspnea	9 (9.9)	3 (10.0)	1 (11.1)	2 (11.1)	1	6 (9.8)	1
Headache	57 (62.6)	24 (80.0)	8 (88.9)	14 (77.8)	1	33 (54.1)	0.243
Body aches	47 (51.6)	19 (63.3)	9 (100)	10 (55.6)	0.261	28 (45.9)	0.666
Watery diarrhea	11 (12.1)	4 (13.3)	2 (22.2)	2 (11.1)	1	7 (11.5)	1
Nausea	2 (2.2)	0 (0)	0 (0)	0 (0)	NA	2 (3.3)	NA

The value is shown in a number format (percentage). NA: not applicable. *p*-values were conducted using Pearson’s Chi-squared test or Fisher’s exact test and adjusted for multiple comparisons using the Benjamini and Hochberg procedure; * 3 disease episodes with a redondovirus detected, but no PCR sequence was obtained for species identification; # between column “Total” of “Redondoviruses positive” vs. column “Redondoviruses negative”.

**Table 6:** Codetection of redondoviruses and other viruses in the respiratory samples analyzed in this study.

	Redondoviruses Positive *				Redondoviruses Negative	*p*-Value #
	Total	Brisavirus	Vientovirus	*p*-Value		
	33	9	23	NA	25	NA
Gemycircularvirus VIZIONS-2013 ^^^	8 (24.2)	2 (22.2)	5 (21.7)	1	7 (28)	0.7
Cyclovirus VIZIONS-2013	4 (12.1)	1 (11.1)	3 (13)	1	5 (20)	0.5
Rhinovirus	4 (12.1)	0 (0)	4 (17.4)	0.3	1 (4)	0.4
Respiratory syncytial virus A	2 (6.1)	0 (0)	2 (8.7)	1	0 (0)	0.5
Statovirus VIZIONS-2013	2 (6.1)	1 (11.1)	1 (4.3)	0.5	0 (0)	0.5
Statovirus	2 (6.1)	0 (0)	2 (8.7)	1	0 (0)	0.5
Enterovirus	1 (3)	0 (0)	1 (4.3)	1	1 (4)	1
Influenza A virus	1 (3)	1 (11.1)	0 (0)	0.3	0 (0)	1
Metapneumovirus	1 (3)	0 (0)	1 (4.3)	1	0 (0)	1
Gemycircularvirus	1 (3)	0 (0)	1 (4.3)	1	0 (0)	1
Coronavirus OC43	0 (0)	0 (0)	0 (0)	NA	1 (4)	0.4

* Number of participants who got infected with redondoviruses at least once during the entire study. NA: not applicable. The value is shown in a number format (percentage). *p*-values were calculated using Pearson’s Chi-squared test or Fisher’s exact test. The *p*-values were adjusted for multiple comparisons using the Benjamini and Hochberg procedure; # by *t*-test. ^ 1 sample with a redondovirus detected, but no PCR sequence was obtained for species identification. # between column “Total” vs. column “Redondoviruses negative”.

## Data Availability

Not applicable.
